# Minimizing the Impact of the COVID-19 Epidemic on Oncology Clinical Trials: Retrospective Study of Beijing Cancer Hospital

**DOI:** 10.2196/26799

**Published:** 2021-03-02

**Authors:** Zhiying Fu, Min Jiang, Kun Wang, Jian Li

**Affiliations:** 1 Beijing Institute for Cancer Research Beijing Cancer Hospital Beijing China

**Keywords:** COVID-19, clinical trials, management strategy, information technology

## Abstract

**Background:**

In view of repeated COVID-19 outbreaks in most countries, clinical trials will continue to be conducted under outbreak prevention and control measures for the next few years. It is very significant to explore an optimal clinical trial management model during the outbreak period to provide reference and insight for other clinical trial centers worldwide.

**Objective:**

The aim of this study was to explore the management strategies used to minimize the impact of the COVID-19 epidemic on oncology clinical trials.

**Methods:**

We implemented a remote management model to maintain clinical trials conducted at Beijing Cancer Hospital, which realized remote project approval, remote initiation, remote visits, remote administration and remote monitoring to get through two COVID-19 outbreaks in the capital city from February to April and June to July 2020. The effectiveness of measures was evaluated as differences in rates of protocol compliance, participants lost to follow-up, participant withdrawal, disease progression, participant mortality, and detection of monitoring problems.

**Results:**

During the late of the first outbreak, modifications were made in trial processing, participant management and quality control, which allowed the hospital to ensure the smooth conduct of 572 trials, with a protocol compliance rate of 85.24% for 3718 participants across both outbreaks. No COVID-19 infections were recorded among participants or trial staff, and no major procedural errors occurred between February and July 2020. These measures led to significantly higher rates of protocol compliance and significantly lower rates of loss to follow-up or withdrawal after the second outbreak than after the first, without affecting rates of disease progression or mortality. The hospital provided trial sponsors with a remote monitoring system in a timely manner, and 3820 trial issues were identified.

**Conclusions:**

When public health emergencies occur, an optimal clinical trial model combining on-site and remote management could guarantee the health care and treatment needs of clinical trial participants, in which remote management plays a key role.

## Introduction

COVID-19 was recognized as a global pandemic on March 11, 2020 [1,2]. Efforts to stem the spread of SARS-CoV-2 led to isolation measures and travel restrictions that have severely hindered the smooth conduct of clinical trials [3]. Participants may no longer be able to visit the hospital to receive treatment [4], resulting in protocol deviations. The regular monitoring and auditing of trials may also be affected [5], leading trials to be postponed or terminated; this in turn can delay marketing of drugs and even lead to the scrapping of development plans for new drugs. Several studies from several countries have reported obvious decreases in the numbers of participants newly enrolled in trials, and many oncology clinical trials were suspended in April 2020 in several countries [6-8].

It seems likely that as COVID-19 outbreaks repeatedly occur in many countries, clinical trials will continue to be conducted under public health emergency conditions for some time to come. The conduct of oncology clinical trials will be particularly challenging, given that such trials usually require many follow-up visits and sophisticated monitoring of complex disease courses [9,10]. Therefore, insights into how to optimize oncology clinical trial management during public health emergencies are urgently needed.

This study examined the experiences of Beijing Cancer Hospital in its efforts to conduct anticancer drug trials through two COVID-19 outbreaks in 2020. During the first outbreak from February to April 2020, the hospital implemented a series of modifications in participant management and monitoring, including remote drug administration, remote visits, and remote trial monitoring. Relying on data processing and application platform (DPAP) technology, remote trial monitoring enables clinical research associates and inspectors to monitor trial data at any time and in any place, regardless of geographical and time constraints. At present, remote trial monitoring is rare in China and other countries. Here, we analyzed how the COVID-19 epidemic affected ongoing clinical trials and whether the hospital’s modifications to trial management during the epidemic helped ensure the smooth conduct of these trials through the second outbreak from June to July 2020. Our experiences may be relevant to other countries as they seek to ensure the functioning of clinical trials during the COVID-19 epidemic and other public health emergencies.

## Methods

This study was approved by the Ethics Committee of Beijing Cancer Hospital, which waived the requirement for informed consent for the participants because those individuals had already provided written informed consent for their medical data to be analyzed and published in an anonymized format for medical research purposes.

### Study Assumption

During public health emergencies, combining on-site and remote clinical trial management models could guarantee the health and treatment needs of clinical trial participants.

### Study Design

During the COVID-19 epidemic, the ultimate goal of the management measures was to ensure the treatment of the participants and the implementation of the trials while avoiding the spread of the disease. In this case, remote management was the determinant of strategy effectiveness. Therefore, in addition to the COVID-19 infection rate of the participants, we selected the indicators that were important to the trials and the participants: protocol compliance rate, rate of loss to follow-up, rate of participant withdrawal, rates of disease progression and mortality, and detection rate of monitoring problems.

Clinical trials that were ongoing at the hospital from February 1, 2020, were eligible to be enrolled in the study. The following data were collected through July 31, 2020: numbers of outpatient and inpatient visits, numbers of enrolled participants, visits that were not performed according to protocol, participants who were not administered according to protocol, losses to follow-up, withdrawals, cases of disease progression, and deaths. These data allowed us to assess the clinical trials during the first COVID-19 outbreak from February to April 2020 and during the second outbreak from June to July 2020. The corresponding data during the periods from February to April 2019 and June to July 2019 were also collected. Data were extracted from the hospital information system (HIS) and clinical trial management system (CTMS). We also collected data regarding the use of the remote monitoring system from February 01, 2020, to July 31, 2020, including the frequency of use, number of trials checked, and number of participants checked.

### Statistical Methods

To assess the impact of the COVID-19 epidemic on the conduct of clinical trials at our hospital, we compared the trial data from February to April 2020 and June to July 2020 with data from February to April 2019 and June to July 2019, respectively. To assess the effects of our hospital’s modifications to the clinical trials after the first COVID-19 outbreak, we compared the trial data obtained during February and July in 2020. All statistical analyses were performed using SPSS, version 22.0 (IBM Corporation). Categorical data were reported as frequencies or percentages, and differences were assessed for significance using the chi-square test.

## Results

### Impact of the COVID-19 Outbreak on Clinical Trials at Beijing Cancer Hospital

**Figure 1:**
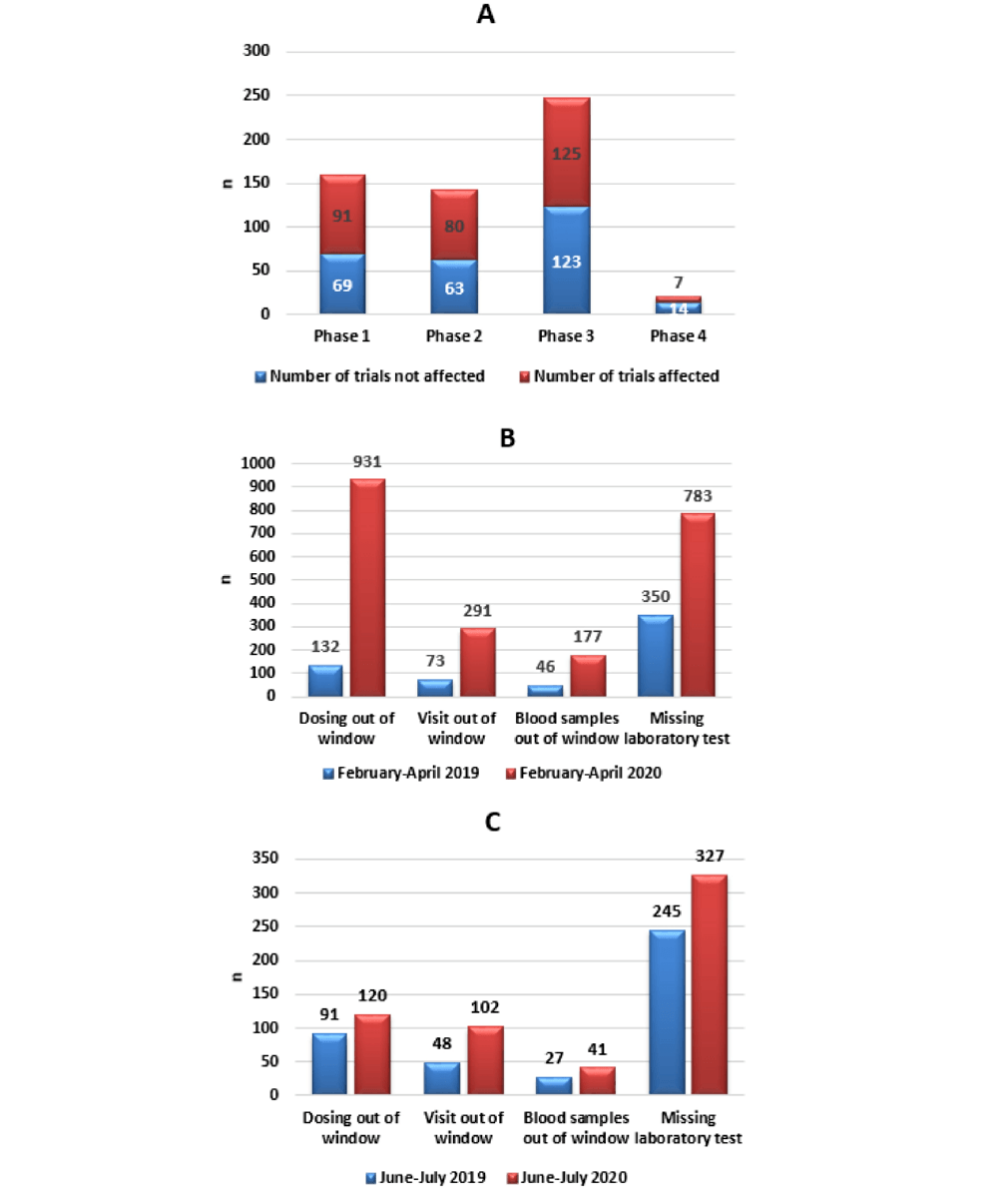
The impact of the COVID-19 outbreak on clinical trials at our hospital. (A) Distribution of product phases affected by the outbreak. (B) Protocol violations from February to April 2020 compared to February to April 2019. (C) Protocol violations from June to July 2020 compared to June to July 2019.

On July 31, 2020, 572 trials of investigational drugs were being conducted at Beijing Cancer Hospital. More than 50% of all trials (303/572, 53.0%) were affected by the COVID-19 outbreak, including 65 international trials and 238 domestic trials. Of the 303 affected trials, 91 were phase 1 trials, accounting for 56.9% (91/160) of ongoing phase 1 trials. Only 28 new patients were enrolled each month through the two outbreaks, which was 6 times fewer than the monthly enrollment in 2019, and the numbers of various types of protocol deviations were higher than for the same period in 2019. The most frequent deviations were “dosing out of window” and “visit out of window.” Due to the outbreak, 42 participants withdrew from trials and 39 were lost to follow-up. The impact of the COVID-19 outbreak on the clinical trials at our hospital is summarized in Figure 1.

### Modifications to Clinical Trial Management During the First COVID-19 Outbreak

#### Remote Trial Approval and Initiation

Starting in February 2020, the hospital implemented information-communication technology to reduce human interactions during clinical trials. The hospital connected its CTMS to an external network so that staff could conduct numerous trial-related activities on the web, including trial review, application for ethics approval, review of collaboration agreements to conduct trials, quality control, and other filings. Trial sponsors and contract research organizations were able to review trials using the same system. Software was also developed to allow our hospital and principal investigators to initiate new trials if justified.

#### Remote Trial Monitoring

**Figure 2:**
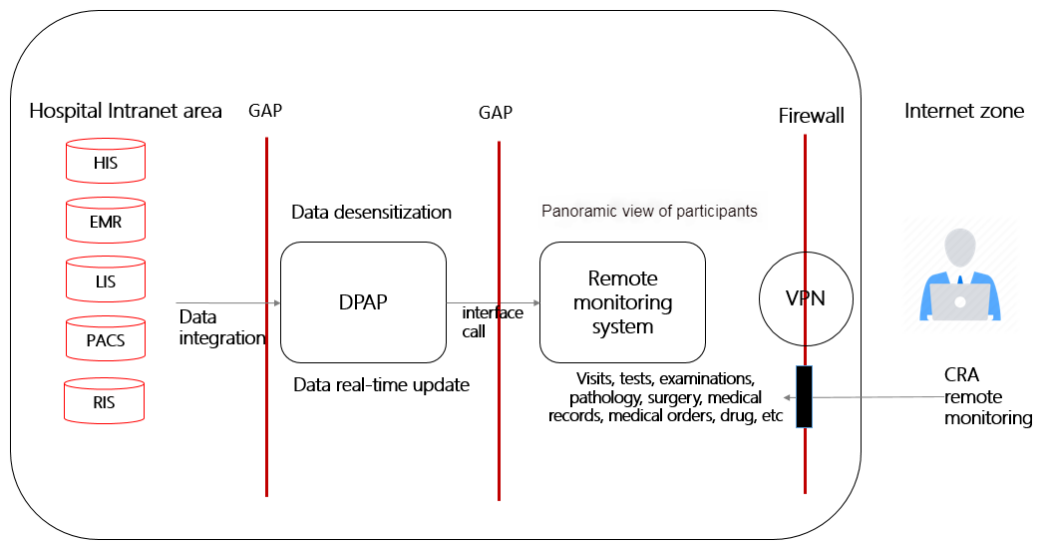
The remote monitoring technology roadmap. CRA: clinical research associate; DPAP: data processing and application platform; EMR: electronic medical record; GAP: gatekeeper; HIS: hospital information system; LIS: laboratory information system; PACS: picture archiving and communication system; RIS: radiology information system; VPN: virtual private network.

**Figure 3:**
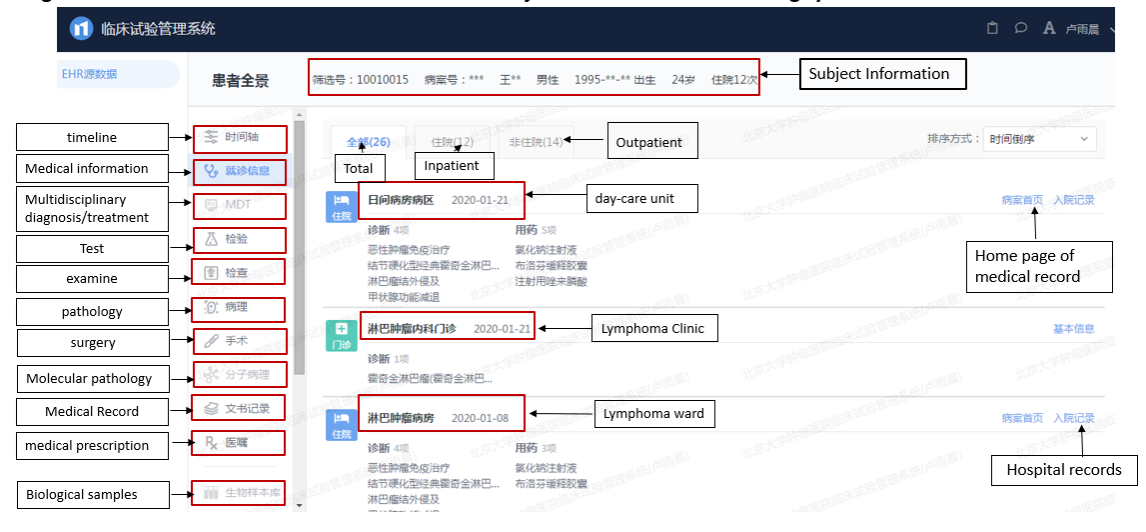
Screenshot of the panoramic view of a clincial trial participant in the remote monitoring system.

A remote monitoring system, relying on DPAP technology, integrating various business systems of the hospital, such as the HIS, electronic medical record, laboratory information system, picture archiving and communication system, and radiology information system, was implemented to allow monitors to conduct virtual “site visits” under a virtual private network (VPN). Monitors could check all the medical treatment data of the participants in hospital as authorized and receive a panoramic data view of the participants under the dimensions of medical treatment, examination, medical records, and medical orders. Figure 2 shows the remote monitoring technology roadmap, and Figure 3 shows a screenshot of a panoramic view of a participant’s data in the remote monitoring system.

#### Remote Visits and Treatment of Participants

Trial participants who were able to return to hospital upon the resumption of trials were tested for SARS-CoV-2 infection based on nucleic acid detection. Hospitalized participants were examined for COVID-19 symptoms by chest computerized tomography. Beds were placed with sufficient distancing.

Trial participants who could not return to hospital were “visited remotely”: the principal investigator of the trial was located an appropriate hospital that was relatively close to the participant’s residence. Priority was given to hospitals that were also participating in the trial, followed by hospitals that had been certified by the National Medical Products Administration to be following good clinical practice [11]. Trial investigators remotely reviewed examination results through email or fax, and they generated the corresponding medical records, which were stored on a cloud-based platform from July 2020.

Trial medications for oral use were sent to these participants if the trial investigator judged that the drug treatment could continue based on the participant’s examination results. Medications were sent by mail using the hospital’s standard operating procedures for mailing of investigational products during major public health emergencies. The participants sent back relevant data by mail.

**Figure 4:**
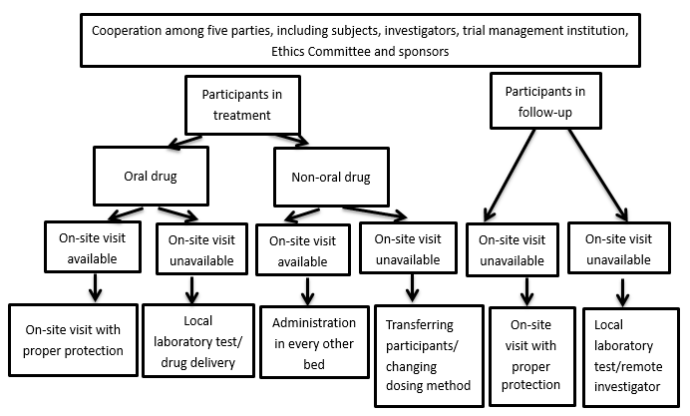
Schedule of the panoramic view of a clinical trial participant's visits and treatments according to modified clinical trial management procedures implemented from February 01, 2020.

In some cases, trial participants originally scheduled for intravenous drug administration were switched to oral administration of an equivalent medication (eg, etoposide) to allow remote drug treatment. These protocol changes were enacted only after discussion among the trial investigators and ethical approval from the hospital. If the originally scheduled intravenous drug administration could not be switched to oral administration, participants received the trial medications intravenously at an appropriate local hospital (see above), after local staff had been trained by trial personnel from Beijing Cancer Hospital. Participants’ medical records were transferred, as necessary, from Beijing Cancer Hospital to the local hospital. Figure 4 depicts the pathway for remote treatment of trial participants.

### Comparison of Clinical Trial Data Between the First and Second COVID-19 Outbreaks

During the first outbreak from February 1 to April 30, 2020, 18 new trials were remotely initiated, and 45 participants were newly enrolled. Moreover, during the second outbreak from June 1 to July 31, 2020, 56 new trials were remotely initiated and 103 participants were newly enrolled, which are nearly double the numbers of trials and enrollments in the first outbreak. By July 31, 572 trials for investigational drugs involving 3718 participants were ongoing at Beijing Cancer Hospital. Between February and July 2020, no infections were recorded among participants or trial staff, and no major procedural errors occurred.

**Figure 5:**
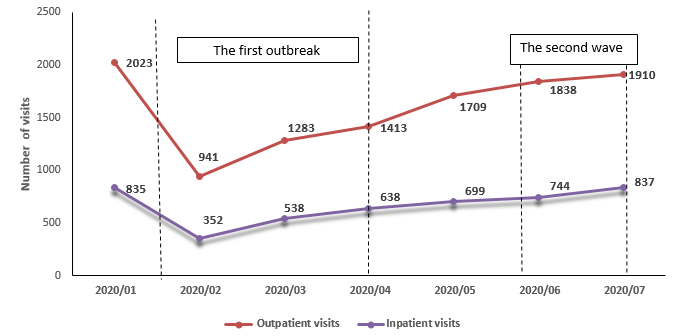
Outpatient and inpatient status of clincial trials at Beijing Cancer Hospital during the COVID-19 epidemic, January 2020 to July 2020.

Analysis of the numbers of visits by inpatients and outpatients to Beijing Cancer Hospital for participation in clinical trials showed that both types of visits decreased in February, coinciding with the first COVID-19 outbreak (Table 1). Thereafter, both types of visits continuously increased and had nearly returned to pre-epidemic levels by July (Figure 5).

**Table 1:** Data on participant visits within clinical trials during the two COVID-19 outbreaks in 2020.

Category	Monthly average for the indicated period	
	First outbreak (February to April)	Second outbreak (June to July)
Return visits to hospital for outpatient examination or treatment	1212	1709
Hospitalizations	509	791
Remote visits	340	109
Drug shipments to participants	281	97
Participants changed from intravenous to oral administration	10	2
Participants transferred to another hospital (times)	16	6
Internet-based diagnostic visits and treatments	0	165
Completed visit/medication (times)	2368	2879
Total required visits/medication (times)	3097	3169

During the latter part of the first outbreak, 1941/9291 visits with participants (20.89%) were conducted remotely, oral medications were mailed directly to participants, or the participants were treated intravenously at carefully selected local hospitals. The protocol compliance rate was 76.46%. During the second outbreak (June to July), the protocol compliance rate was significantly higher at 90.85% (2879/3169, *P*<.001) (Table 2). Loss to follow-up was significantly smaller during the second outbreak than during the first (7/3718, 0.19% vs 32/3570, 0.89%, *P*<.001), as was participant withdrawal (6/3718, 0.16%, vs 36/3570, 1.00%, *P*<.001). In contrast, the rates of disease progression and mortality did not differ statistically between the two outbreaks (Table 2). Thus, the clinical trials at our hospital remained stable across both COVID-19 outbreaks, and the rate of compliance for the entire period was 85.24% (16007/18778).

During the whole outbreak period, the infection rate of SARS-CoV-2 among personnel involved in clinical trials was 0, and the error rate in the clinical trials was 0.

**Table 2:** Clinical trial outcomes.

Outcome	Value, n/total (%)		*P* value
	First outbreak	Second outbreak	
Protocol compliance	2368/3097 (76.46)	2879/3169 (90.85)	<.001
Loss to follow-up	32/3570 (0.89)	7/3718 (0.19)	<.001
Withdrawals	36/3570 (1.00)	6/3718 (0.16)	<.001
Disease progression	88/3570 (2.34)	88/3718 (2.37)	.86
Mortality	48/3570 (1.33)	68/3718 (1.83)	.08

### Effects of the Measures on Trial Quality During the COVID-19 Epidemic

By July 31, 2020, 176 clinical research associates from 76 sponsors or clinical research organizations had used the remote monitoring system to monitor 1318 participants in 228 trials conducted in 16 departments of Beijing Cancer Hospital. The total number of log-ins to the system was 10,470, and the median number of monitoring visits was 23 (range 1-729). The total number of issues logged in the remote monitoring system was 3820, corresponding to an average of 16.75 per trial and 2.90 per participant. The most frequent findings were errors or omissions on case report forms (950/3820, 24.87% of all issues), lack of compliance with planned trial visits (849/3820, 22.23% of all issues), and lack of compliance with protocol administration (713/3820, 18.66% of all issues). The rate of findings with remote monitoring between original records, informed consent, adverse events, and investigational drugs in 2020 were significantly lower than the rate during the same period in 2019 (*P*<.001), but the two monitoring approaches were similar in terms of case report forms, concomitant medication, and biological samples (*P*>.05) (Table 3).

**Table 3:** Clinical trial–related events detected during on-site monitoring in 2019 and remote monitoring in 2020.^a^

Error or omission in a trial event	Value per capita, n (%)		*P* value
	Remote monitoring in 2020 (n=1318)^b^	On-site monitoring in 2019 (n=1120)^c^	
Case report forms	950 (72.08)	805 (71.88)	.91
Visits not conducted per protocol	849 (64.42)	579 (51.69)	<.001
Drug not dosed per protocol	713 (54.10)	279 (24.91)	<.001
Adverse events	513 (38.92)	643 (57.41)	<.001
Concomitant medications	435 (33.00)	401 (35.80)	.15
Original record	154 (11.68)	874 (78.04)	<.001
Biological samples	146 (11.08)	159 (14.20)	.02
Other drug problems	33 (2.50)	119 (10.63)	<.001
Informed consent	27 (2.05)	119 (10.63)	<.001

^a^Data were compared for the same trials for the period of February to July in 2019 or 2020.

^b^Total errors/emissions=3820; 289.83% cumulative for all events.

^c^Total errors/emissions=3978; 355.18% cumulative for all events.

## Discussion

The COVID-19 outbreak is a major global public health emergency, and at its beginning, authorities were taken by surprise [12]. Our analysis of clinical trials at Beijing Cancer Hospital from the beginning of the COVID-19 epidemic showed that 53.0% of investigational drug trials were affected, particularly phase I clinical trials, which involve more extensive interventions. During the first outbreak (February to April 2020), the rate of “dosing out of window” was 6 times higher than the rate during the same period in 2019, while the rate of “visits out of window” was 3 times higher than in 2019. This is especially true of clinical trials of anticancer drugs, which usually require drug administration every 1-2 weeks, and most trials require regular sample collection and patient examinations. These protocol deviations could severely compromise the quality of the trial data as well as the safety and interests of participants [13].

While regulatory authorities in the United States, European Union, and China as well as other associations have guidelines for clinical trial conduct and management during the COVID-19 epidemic [14-17], implementing them in hospitals is not always straightforward. At Beijing Cancer Hospital, discussions were conducted among trial participants, trial investigators, members of the hospital’s Ethics Committee, and trial sponsors to develop modifications to the standard management of the clinical trials and participant visits to protect the health and safety of participants as well as the integrity of the trial. These discussions and the ensuing measures were documented in real time and archived at the hospital and in the records of the Ethics Committee.

Starting from the middle of the first COVID-19 outbreak, our hospital resumed clinical trials with a series of measures to reduce virus transmission: diagnostic visits and treatment were conducted by appointment only or via the internet; participants were visited remotely; drugs were mailed to participants, including replacement of intravenous treatments with oral treatments that could be mailed; and participants were sent to local hospitals, protecting them from infection during travel to the clinical trial center. To encourage participants to return to the hospital for treatment during the second outbreak, we subjected them to nucleic acid tests and provided beds with adequate separation. Only 50% of beds in each ward were available to patients. These measures may help to explain why more participants returned to the hospital during the second outbreak than during the first. All these measures led to higher protocol compliance rates during the second COVID-19 outbreak than during the first, as well as lower rates of loss to follow-up and withdrawal.

Internet diagnostics and treatment are a new direction in the medical industry, and our experience suggests that this approach can be effective for conducting clinical trials during public health emergencies. Quality control standards are needed to ensure high-quality treatment comparable to that with in-person medicine. To that end, China and other countries have issued regulations and policies to standardize internet diagnostics and treatment [18-20].

Just as internet-based approaches can bring trial clinicians and participants together safely, remote monitoring can allow trial auditors and sponsors to perform necessary reviews. Already in 2019, the US Food Drug and Administration had encouraged sponsors to use remote monitoring for early detection of problems in clinical trials [21], and several national and other agencies have since encouraged remote monitoring in response to the COVID-19 pandemic [14-17]. At Beijing Cancer Hospital, approximately 40% of clinical trials were remotely monitored from February to July 2020, and these activities identified 16.75 issues per trial. Our experience supports the expanding reliance on remote trial monitoring to safeguard patients and trial quality.

Clinical trials in China and abroad face challenges due to the COVID-19 pandemic [22]. Our experience supports the idea that appropriate software and network infrastructure can allow medical facilities to conduct clinical trials effectively [23-25], while properly archiving, managing, and sharing the large amount of data generated [26]. It may even be possible to bypass some aspects of participant follow-up by using smartphones or other wearable devices that can automatically transmit participant data [27]. Conducting a remote clinical trial depends on having a complete information management system, infrastructure for fast and safe delivery of medicines, and a safe, seamless system for managing and transferring trial data.

Remote trial management at Beijing Cancer Hospital can still be improved. For example, we were unable to use the remote informed mode due to technical limitations. Insufficient personnel and delays in data transmission during the epidemic in 2020 contributed to data errors or omissions on case report forms, which accounted for 24.87% of all issues. The system of remote monitoring and automatic collection of clinical trial data should be improved, particularly the extraction of data from paper records.

This study was necessarily retrospective, which increases the risk of selection and information bias. As the pandemic continues, it may be advisable to launch relevant prospective studies to assess the efficacy and efficiency of measures to ensure the smooth conduct of clinical trials during a public health emergency.

### Conclusion

Clinical trials have been greatly impacted during the current public health emergency of the COVID-19 pandemic. By using information technology, Beijing Capital Hospital was able to ensure the smooth conduct of hundreds of oncology clinical trials. This success was due to a clinical trial management model combining on-site and remote trial approval, initiation, visits, administration, and monitoring. Our experience provides a reference for clinical trial management under the current pandemic and in future public health emergencies.

## Abbreviations

CTMS: clinical trial management system
DPAP: data processing and application platform
HIS: hospital information system
VPN: virtual private network
